# GenePlexus: a web-server for gene discovery using network-based machine learning

**DOI:** 10.1093/nar/gkac335

**Published:** 2022-05-17

**Authors:** Christopher A Mancuso, Patrick S Bills, Douglas Krum, Jacob Newsted, Renming Liu, Arjun Krishnan

**Affiliations:** Department Of Computational Mathematics, Science and Engineering, Michigan State University, East Lansing, MI 48824, USA; Data Management and Analytics, IT Services, Michigan State University, East Lansing, MI 48824, USA; Data Management and Analytics, IT Services, Michigan State University, East Lansing, MI 48824, USA; Data Management and Analytics, IT Services, Michigan State University, East Lansing, MI 48824, USA; Department Of Computational Mathematics, Science and Engineering, Michigan State University, East Lansing, MI 48824, USA; Department Of Computational Mathematics, Science and Engineering, Michigan State University, East Lansing, MI 48824, USA; Department of Biochemistry and Molecular Biology, Michigan State University, East Lansing, MI 48824, USA

## Abstract

Biomedical researchers take advantage of high-throughput, high-coverage technologies to routinely generate sets of genes of interest across a wide range of biological conditions. Although these technologies have directly shed light on the molecular underpinnings of various biological processes and diseases, the list of genes from any individual experiment is often noisy and incomplete. Additionally, interpreting these lists of genes can be challenging in terms of how they are related to each other and to other genes in the genome. In this work, we present GenePlexus (https://www.geneplexus.net/), a web-server that allows a researcher to utilize a powerful, network-based machine learning method to gain insights into their gene set of interest and additional functionally similar genes. Once a user uploads their own set of human genes and chooses between a number of different human network representations, GenePlexus provides predictions of how associated every gene in the network is to the input set. The web-server also provides interpretability through network visualization and comparison to other machine learning models trained on thousands of known process/pathway and disease gene sets. GenePlexus is free and open to all users without the need for registration.

## Graphical Abstract

**Graphical Abstract:**
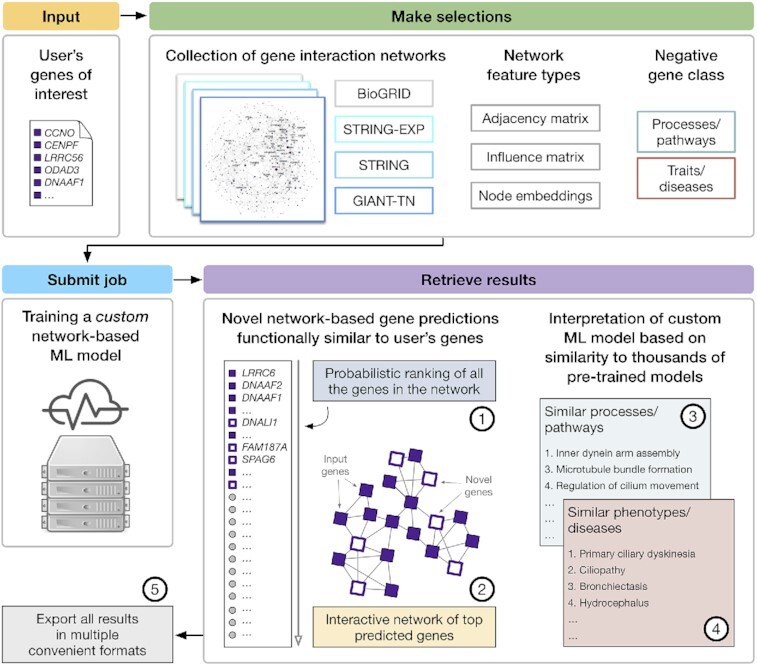
GenePlexus is a cloud-based webserver that enables biomedical researchers to discover novel genes functionally similar to their genes of interest using network-based machine learning.

## INTRODUCTION

Most complex functions, phenotypes, traits and diseases involve complex interactions between many genes. With the advent of high-throughput, high-coverage technologies (1,2), researchers are able to measure various types of signals pertaining to these phenomena on a genome-wide scale and ultimately generate a list of genes of interest. For instance, differential expression analysis (3,4) of bulk- or single-cell transcriptomes allow researchers to generate gene sets of interest, which provide some initial insight into the molecular underpinnings of the experimental factors being studied. However, these gene sets often suffer from a few drawbacks: (i) the gene sets can be incomplete (i.e. containing false negatives) and noisy (i.e. containing false positives) and (ii) the gene list inherently lacks information about how the individual genes interact with each other and with other genes in the genome.

The ability to computationally refine an experimentally-derived gene set by prioritizing genes of interest and predicting other novel genes that may be highly related to the set is a grand challenge in biomedical research (5–11). Although experimental validation is always required, the sheer number of possible novel associations require computational techniques to guide which genes to study next. Over the past few decades, computational methods that incorporate information from genome-wide, context-specific molecular-networks have shown state-of-the-art results (12–21). Recently, we have shown that directly using the connections from genome-wide molecular networks as the features to a supervised machine learning model (referred to as GenePlexus) is a robust, data-driven way to computational predict how associated a gene is to a given input gene set (22).

As powerful as these computational methods can be, their impact is fully realized only if they can be put into the hands of biomedical researchers, regardless of programming and computational background. Publicly available web-servers are a great platform for disseminating these results and an ideal web-server would have the following properties:

- Handle gene sets generated across vastly different biological contexts and from different technologies.
- Allow the user to choose from a suite of molecular networks that best fit the biological question.
- Provide predictive insights about additional genes most functionally similar to the user-supplied gene set and provide a confidence level of these predictions in a timely manner.
- Enable the researcher to interpret the underlying computational model and to visualize the connectivity of the top-ranked genes.
- Provide a user interface that is intuitive and easy to use for a biologist regardless of programming skills, provide extensive help/tutorials, and provide open-source code for the predictive model and web-server.

**Figure 1.**
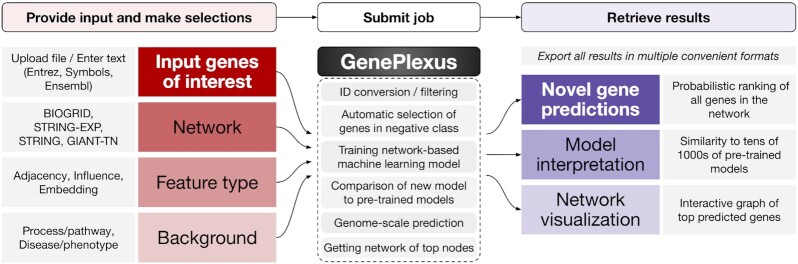
The workflow of the GenePlexus web-server. First the user uploads a gene set of interest and choses the network and representation and how the negative genes should be selected. Next, the data is prepared, the model is trained and the results are created. Finally, the user can retrieve gene predictions, gain insight into the trained model and visualize the network connectivity of the top genes interactively through their browser.

In this work, we present the GenePlexus web-server which addresses all the needs above [Figure 1]. A user can upload a set of genes and choose the desired network properties. Then the web-server trains a *custom* supervised machine learning model using the user-supplied genes as positive labels. Within a few minutes, the user can then retrieve an association probability for every gene in the network, interpret the trained model through a comparison to other models trained on known gene sets that correspond to process/pathways and diseases from the Gene Ontology (23,24) and DisGeNet (25,26) databases, respectively, and visualize the network connectivity of the top-ranked genes. We believe that the GenePlexus web-server will greatly benefit anyone who is looking to determine novel associations to a given gene list in a biologically interpretable manner.

There are a number of comparable web-servers that analyze a user-supplied gene set in the context of a molecular network, but they all have some limitations. Web-servers for networks such as STRING (27) and GIANT (20) do not provide a predictive element, instead focussing on offering an interactive visualization of the gene set within their networks. Web-servers such as GeneMania (12), HumanNet (28), ToppGene (29) and MaxLink (30) provide predictions using the method of label propagation, a semi-supervised method which our model has been shown to outperform (22). DGLinker (31) is a powerful and comprehensive web-server that trains a supervised machine learning model on the user-supplied gene set. However, the supervised learning model in DGLinker uses three features that are mined from a vast amount of data sources. In comparison, GenePlexus uses the entirety of the network connections as input to the machine learning model. Additionally, DGLinker is specific to gene-disease discovery, whereas GenePlexus is task agnostic.

A key feature that sets GenePlexus apart is how model interpretation is implemented. All the web-servers mentioned above only offer (if offered at all) insights about the input gene set and, possibly, the highly associated novel genes, through the widely-used analysis technique of gene set enrichment. As there exist many excellent open-source tools for enrichment analysis, we chose not to implement that on GenePlexus. Instead, we provide interpretation of the custom trained machine learning model. We do this by comparing the model trained on the user-supplied gene set to thousands of models that were trained on known biological processes and pathways in the Gene Ontology or diseases in DisGeNet.

## WEB-SERVER WALKTHROUGH

The main purpose of the GenePlexus web-server is to discover novel genes that are functionally similar to a user defined set of ‘genes of interest’. This purpose is accomplished by training a molecular-network-informed machine learning model specific to the user-supplied genes. The key features of the web-server are:

- Prediction of how every human gene is functionally similar to the user-supplied gene set.
- Allow the user to choose between a number of different molecular networks, varying in interaction source, coverage, and density. The user can also choose how the networks are represented in the machine learning model.
- Interpretation of the model by comparing the model trained using the user-supplied gene set to thousands of models pre-trained using gene sets annotated to biological processes in the Gene Ontology and diseases in DisGeNet.
- Visualization of the network connections for the top-ranked genes.
- The web-server is open source (https://github.com/krishnanlab/geneplexus_app) and has extensive help documentation, both in the form of a help page as well as video tutorials.

## INPUTS

### Adding genes

**Figure 2.**
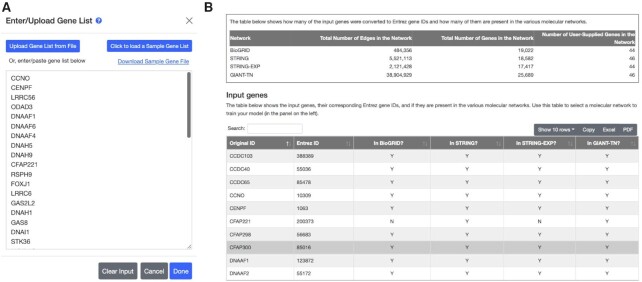
Uploading and validating the gene set. (**A**) The user can either paste gene IDs or upload them from a file. (**B**) Upon clicking the Done button, the genes are converted in Entrez ID space and the overlap of the gene set with the genes in each network is displayed.

The first step is for the user to add a set of human genes [Figure 2A]. Users can do this by either entering the genes manually into a box or uploading a file. The genes can be identified using Ensembl IDs (ENSG, ENSP or ENST), Gene Symbols or NCBI Entrez IDs. The web-server uses cookies to allow the added genes to persist as the user navigates across the pages and to allow the user to edit or add additional genes manually or with a file. We emphasize that no tracking cookies are utilized.

### Validating genes

The user will then click the ‘Done’ button, which will first convert the user-supplied genes into Entrez ID space. This is done because all the networks, gene set collections, and pre-trained models are in Entrez ID space. Next, the web-server determines which of the input genes are present in the different molecular networks and returns this information as a brief summary in the form of a searchable, downloadable table [Figure 2B].

## SELECTING PARAMETERS FOR SETTING UP MACHINE LEARNING MODEL

The user will then select a few parameters that will be used to train the model and generate the results:

- **Molecular Network**: The user can choose from four different human networks [Table S1] that vary in interaction source, coverage, and density (BioGRID (v4.2.191) (32,33), STRING (v11.0) (27), STRING-EXP (v11.0) (27) and GIANT-TN (v 1.0) (20)).
- **Feature Type**: This is how the connections in the network are represented in the machine learning model (Adjacency, Influence, Embedding).
- **Negative Gene Class**: This is a gene set collection [Table S2] that will be used to select negatives based on whether the input genes correspond to a process/pathway or a disease.

For more information about these choices, see the ‘Running the model’ section and the *Supplemental Material*. Additionally, the Help page of the web-server provides some guidance on which choice of parameters would be appropriate for a given user-supplied gene set.

The user also has the *option* to provide two additional pieces of information:

- **Jobname**: GenePlexus automatically creates an eight digit random alpha-numeric job name. In addition, the user is able to supply a memorable prefix to this name.
- **Email**: The user can add their email address to receive a message when the job has started and when the job is completed. These emails include the job parameters and the url to retrieve the job results.

## RUNNING THE MODEL

When the user hits the ‘Submit’ button, they will be directed to the jobs page where they can see the status of all recent jobs that were submitted. Once the job is completed, the link to display the results will become active. After the job is submitted, the user's gene set and selected parameters will be passed to the computational model that performs the following steps

- **Positive and negative gene selection**: The positive examples in the machine learning model are all the genes in the user-supplied gene list that could be mapped to Entrez IDs and were present in the chosen network. The negative examples are chosen in the following way:
  - Consider all genes that are present in the selected gene set collection (‘Negative Gene Class’) as negative examples.
  - Remove from this negative set any genes in the positive set.
  - Additionally, remove genes annotated to gene sets in the collection that significantly overlap with the positive set.
- **Training a machine learning model**: The machine learning model is a regularized (l2-norm) logistic regression model with a regularization parameter of 1. The features used in the model are created based on the user's selection of the network and feature type, where the feature types are:
  - **Adjacency**: Features are the connections in the original network.
  - **Influence**: Features are generated by using a random-walk with restart diffusion kernel on the original network.
  - **Embedding**: Features are generated using the node embedding algorithm *node2vec* (34).
- **Evaluating the machine learning model**: If the positive set contains at least 15 genes, the model is internally evaluated using 3-fold cross validation. The cross validation results provide a very useful measure of whether the model worked well on the gene set of interest, and can be used to help a user choose the optimal network, feature, and negative selection options.
- **Generation of the results**: Once the model is trained, it is used to predict a score for every gene in the network. The results also include the similarity of thousands of pre-trained process/pathway- and disease-gene models to the custom-trained model, and a visualization of the network connectivity of the top-ranked genes.

For more detailed information on the machine learning model see (22) and the *Supplemental Material*.

## RESULTS

The GenePlexus web-server returns a number of useful results that can either be downloaded as one compressed (zip) file or individually in multiple useful formats. The results page can be navigated through a number of tabs, and the parameters used to generate the results as well as the cross-validation results are displayed at the top of each tab.

### Predicted gene associations

The main result returned is the predicted functional similarity of every gene contained in the selected network to the user-supplied input list. The first three columns give details on the genes with hyperlinks to NCBI gene pages with more information. The last four columns provide the following information:

- **Probability**: The predicted probability from the logistic regression model.
- **Training-Label**: The label of the gene used during training with P: positive, N: negative and U: unused.
- **Known/Novel**: Positive genes are considered ‘Known’ and negative genes and genes unused during training are considered ‘Novel’.
- **Rank**: Provides the ranking of the gene based on its predicted probability.

These results are returned as a searchable table that is originally sorted by predicted probability. For example, the user can display just the scores for the ‘Novel’ genes by typing ‘Novel’ into the search box above the table.

### Model interpretability

In web-servers that provide a similar service, if interpretability of the results is offered, it is done so in the form of biological processes (or other curated gene sets) enriched in the user-supplied gene set with the addition of predicted genes. As gene set enrichment is easily accessible through dozens of web-servers and software packages, we instead provide the user with some interpretation of the custom-trained machine learning model.

We accomplish this by comparing the model trained on the user-supplied gene set to thousands of models pre-trained using known gene sets corresponding to biological processes from the Gene Ontology and diseases from DisGeNet. These pre-trained models are built using the same network, feature, and negative gene set collection used to train the custom model trained on the user-supplied gene set. For detailed information on how the similarity score is calculated, see the *Supplemental Material*. We highlight that this feature is unique to GenePlexus. It provides a very network-specific interpretation of the trained model that relies on data generated using >10 000 computational hours on high-memory nodes.

There are two tabs that show the most similar models trained on biological process (Gene Ontology) and disease (DisGeNet) gene sets. In each tab, the first two columns in the table are the ID and long-form name of the known gene set along with a hyperlink to more information. The last two columns contain the similarity to the user's custom-trained model and the rank.

The tables that contain the predicted gene association scores or the similarity to pre-trained models only include the top 500 entries. This is done to increase the speed in which the user can load and interact with these tables. At any point, the user can easily download the full results, which contain >17k gene predictions and similarities to thousands of known gene sets.

### Network graph

Finally, the top gene predictions are also visualized in the context of the original network that was used to train the model. The user can directly change the number of nodes (up to a maximum of 50) or view nodes based on a prediction probability threshold. The set of edges that are displayed can also be changed by setting an edge weight threshold. Individual nodes can be dragged to specific positions and the entire network can be panned and zoomed. Upon clicking on a node, a list of information about that node is supplied.

## WEB-SERVER IMPLEMENTATION

The web-server is implemented using services on the Microsoft Azure cloud platform. The front end is a low resource service that allows the user to upload genes and select parameters, and was written using the microframework Flask. When a job is submitted, the web-server automatically creates a high-resource containerized instance in Azure that contains a Docker version of the source code needed to train the model and generate the results. Once the job is complete, this container is automatically deleted. The ability of the web-server to automatically create and delete these high-resource containers on-demand allows it to simultaneously train numerous machine learning models at minimal costs.

A key feature of the GenePlexus web-server is the ability to allow a user to choose from a variety of networks. Although the networks contained in the current version vary greatly in interaction source, coverage, and density, our implementation can add new networks based on user feedback. We have designed the backend data formats and structure to be flexible so that it is easy to incorporate new networks and gene set collections.

## BENCHMARKING THE GENEPLEXUS METHOD

The supervised network-based machine learning model that forms the backbone of GenePlexus has been extensively benchmarked in (22). In that work, the supervised model was shown to outperform the widely-used, state-of-the-art method of label propagation (12–15,35–48). The comparison included a number of different tasks (function-, disease-, trait-gene prediction), networks (BioGRID, STRING, InBioMap, GIANT-TN, STRING-EXP), validation schemes (temporal holdout, study-bias holdout, 5-fold CV), and evaluation metrics (auPRC, P@topK, auROC).

## ILLUSTRATIVE EXAMPLE

Throughout this work, we demonstrate the utility and features of GenePlexus by applying it to discover genes associated with primary ciliary dyskinesia (PCD). PCD is a genetic condition in which the microscopic organelles (cilia) in the respiratory system have defective function. While a few genes associated with PCD are already known, the genetic cause of the disorder is unknown in many individuals with PCD, making it critical to continue identifying novel PCD genes. Here, we used GenePlexus to predict novel genes associated with PCD based on a gene interaction network, starting with a set of 46 known PCD genes, obtained from the DisGeNet database. The PCD model was trained using the adjacency matrix representation of the STRING network, and the negative genes were determined based on other similar diseases in DisGeNet. This is the same example gene set available to a user to explore on the GenePlexus web-server.

**Figure 3.**
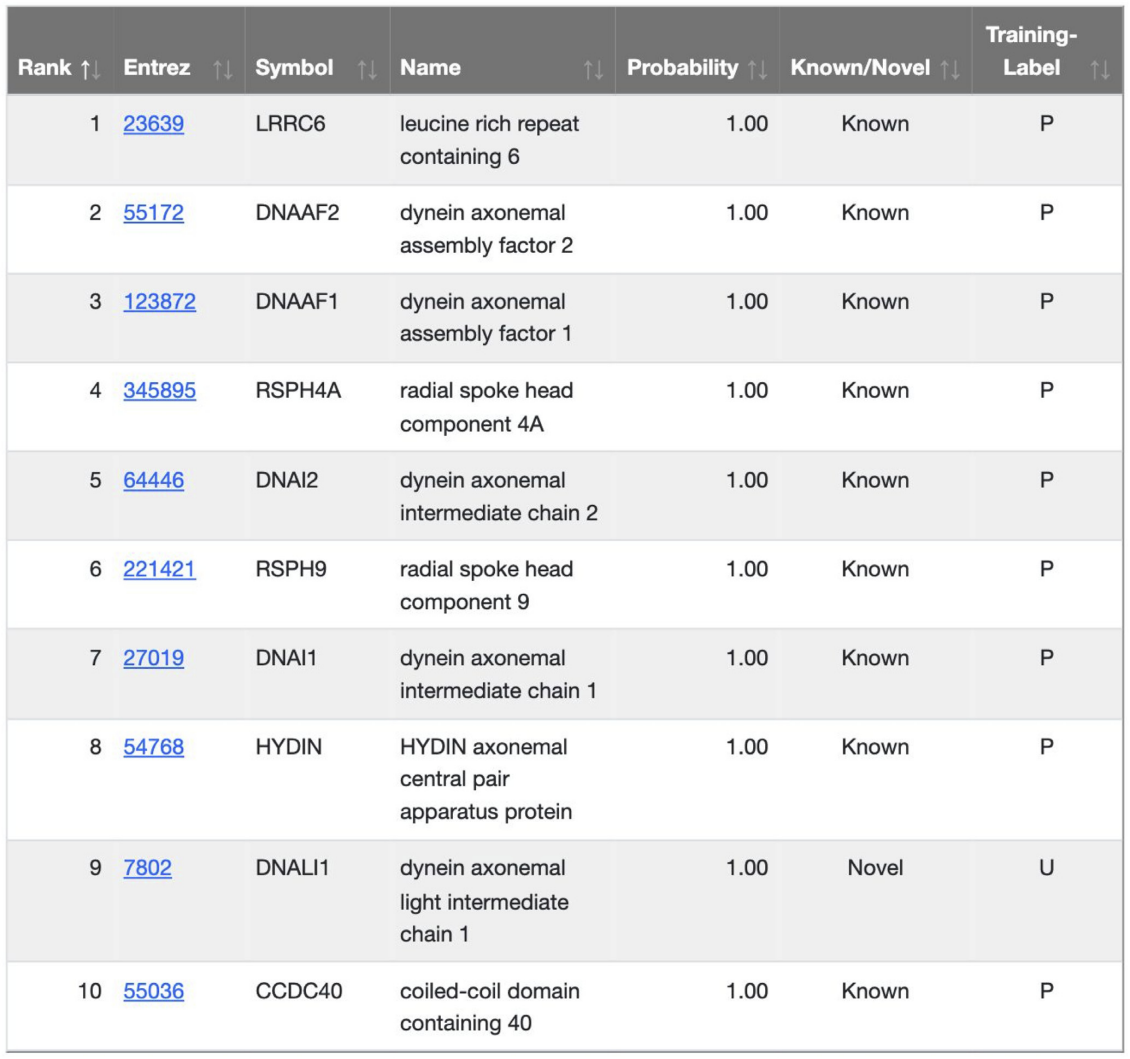
Genome-wide Prediction. For every gene in the genome-scale molecular network that was used to train the model, a score is calculated of how associated it is to the user-supplied gene set and displayed as an interactive table.

**Figure 4.**
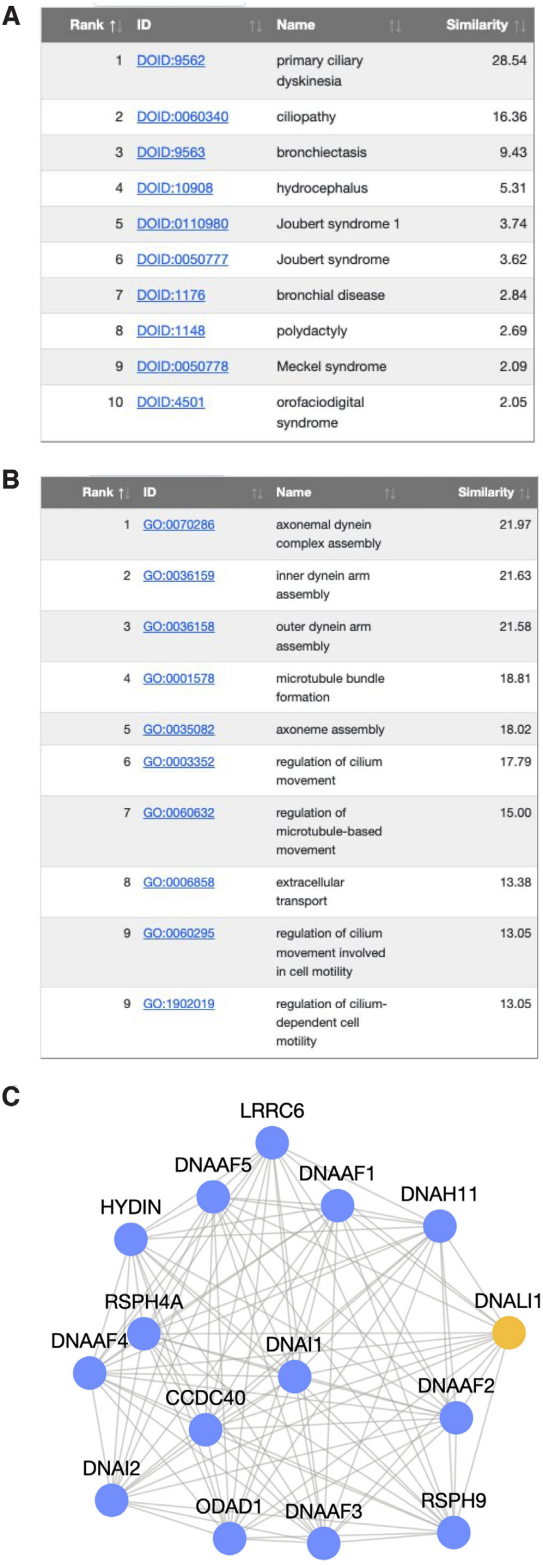
Interpretability features of GenePlexus. (A) The model trained using the user-supplied gene set is compared to thousands of models pre-trained on known gene sets from the (**A**) GeneOntology and (**B**) DisGeNet databases. (**C**) The network connectivity of the top associated genes are displayed as an interactive graph.

Typically in the GenePlexus web-server, as expected, the top-ranked genes consist of many genes included in the user-supplied gene set, and this can be seen for PCD [Figure 3]. A number of these genes belong to the family of axonemal dyneins that cause sliding of microtubules in the axonemes of cilia and flagella (49,50). With the above stated network choices, GenePlexus predicts that *DNALI1*, dynein axonemal light intermediate chain 1, is functionally similar to this input set and is highly connected to known positive genes in the network [Figure 4C]. Additionally, there exists experimental evidence that *DNALI1* is associated with PCD (51,52).

When comparing the user's custom-trained model to models pre-trained on known disease gene sets from DisGeNet [Figure 4A], unsurprisingly the closest models are ‘PCD’ (since this was trained using the same input genes and parameters as the user trained model) and ‘ciliopathy’, which is PCD’s parent term in the Disease Ontology (53). Other top associated diseases are bronchiectasis, hydrocephalus and joubert syndrome, which are other ciliopathy related diseases.

GenePlexus also compares the user's custom-trained model to models pre-trained on known biological process gene sets from Gene Ontology [Figure 4B]. It can be seen that most of the top models are related to either dynein assembly or cilia movement, which have been shown to be underlying mechanisms deregulated in PCD (52). Though the above two analyses may seem similar to gene set enrichment analysis, we note a key difference. Instead of providing interpretation of the list of genes directly, GenePlexus interprets the machine learning model custom-built for PCD to identify diseases and processes that have highly similar patterns of network connectivity compared to PCD. Thus, even if a relevant process/pathway/disease has few (if any) genes overlapping with known PCD genes, it will be considered similar to PCD if the two sets of genes have overlapping network neighborhoods. This model interpretation approach takes advantage of pre-training thousands of models that took >10 000 computational hours, and is an analysis that is unique to GenePlexus.

## DISCUSSION

The GenePlexus web-server provides a powerful tool that any researcher can use to understand and expand any list of human genes generated from an experimental/empirical study including omics profiling, phenotypic assay, association study or drug screen. The web-server leverages the strengths of genome-scale human gene networks and machine learning to help users discover additional novel genes that are functionally similar to their list of input genes. Given a list of input genes, GenePlexus predicts novel genes that have similar network neighborhoods with the input genes, even when these novel genes are not directly connected to the input genes. The web-server achieves this by building a custom machine learning model that finds patterns of connectivity in the network that are distinctive to the input genes and then using this model to find other genes that have similar network patterns. In addition to expanding the original gene set, this analysis illuminates the functional relationships between the known and novel genes by placing them in the context of a molecular network. GenePlexus also helps the user peer into the blackbox by revealing similar machine learning models built for other biological gene sets. We have implemented features so that, at every stage of the analysis, users can export both top-ranked and full results in various convenient formats including plain-text tables, PDFs and image files (as appropriate).

While the GenePlexus web-server contains a number of useful features and options to choose from, we are open to and encourage users to suggest new features. Future versions of the web-server will likely include a larger variety of networks (both human and model species), a public and searchable database of anonymized results from machine learning models trained by other users, the ability to predict novel genes in model species based on human gene networks, and the *option* for users to create accounts to help them keep track of past jobs. We plan on updating the web-server on a yearly basis, which includes adding additional features as well as updating the data used by the web-server. We will continue to have older versions of the web-server publicly available through links on the most current version.

Though many modern web-servers implement an API that allows users to access the web-server programmatically, implementing this feature in a predictive web-server like GenePlexus presents unique challenges. Using an API, a single user could submit many jobs in a short amount of time. Though this could be very useful for a computational biologist looking to expand many gene sets, each submitted job launches a high-resource container on the cloud, which becomes an expensive endeavor. To this end, we have released an open source python package (https://pypi.org/project/geneplexus/) that could be used on its own without the web-server.

## DATA AVAILABILITY

The GenePlexus web-server is freely available at https://www.geneplexus.net/ and the code base for creating the web-server is freely available at https://github.com/krishnanlab/geneplexus_app.

## Supplementary Material

gkac335_Supplemental_File
