# Supplementary material for: GenePlexus: a web-server for gene discovery using network-based machine learning
Source: Nucleic Acids Res. 2022 May 17;50(W1):W358–66. doi: 10.1093/nar/gkac335 (PMC9252732; doi:10.1093/nar/gkac335)
Supplement: gkac335_Supplemental_File [file gkac335_supplemental_file.pdf]

# GenePlexus: A web-server for gene discovery using network-based machine learning

# Supplemental Material

### Section 1: Networks

The networks used on the web-server are BioGRID (1, 2), STRING-EXP (3), GIANT-TN (4), and STRING (3). Detailed information about the network properties and sources can be seen in Table S1, with the network construction method and interaction type information coming from (5). BioGRID (version 4.2.191) is a low-throughput network that includes both genetic interactions, as well as physical protein-protein interactions (1, 2). STRING (version 11.0) is a high-throughput, scored network that aggregates information from many data sources (3). We used two different STRING networks. First, we used the "combined" network that directly includes database annotations, text-mining, ortholog information, co-expression, and experimental determined interactions (referred to as "STRING" on the web-server). We also used a subset of edges in STRING that had just the "experiments" data, thus restricting the network to one constructed just from experimental determined interactions in humans (referred to as "STRING-EXP" on the web-server). For both networks, we used the corresponding relationship scores as edge weights, after scaling them to lie between 0 and 1. The GIANT-TN (version 1.0) network is the tissue-naïve network from GIANT, referred to as the "Global" network on the HumanBase website, and is constructed from both low- and high-throughput data, and includes information from co-expression, non-protein sources, regulatory data, and physical protein-protein interactions (4). The GIANT-TN network is a fully connected, scored network. To add sparsity to the GIANT-TN network, we removed all edges with scores below 0.01 (equal to the prior in the Bayesian model used to construct the network). We used all edge scores (weights) unless otherwise noted, and the nodes in all networks were mapped into Entrez genes using the MyGene.info database (6, 7). If the original node ID mapped to multiple Entrez IDs, we added edges between all possible mappings.

**Table S1. Information on the molecular networks**. LT : low-throughput, HT : high-throughput, G : genetic, P : physical, E : Experimentally determined, DA : database annotations, CE : co-expression, NP : non-protein, R : regulation, TM : text-mining, O : orthologous.

| Network | Number of Genes | Number of Edges | Network Construction Method | Weighted | Interaction Type |
|---|---|---|---|---|---|
| BioGRID | 19,022 | 484,356 | LT | No | G, P |
| STRING-EXP | 17,417 | 2,121,428 | HT | Yes | E |
| GIANT-TN | 25,689 | 38,904,929 | LT, HT | Yes | CE, NP, P, R |
| STRING | 18,582 | 5,521,113 | HT | Yes | TM, CE, O, DA, P |

### Section 2: Network representation

We utilize three distinct representations of molecular networks: the adjacency matrix, an influence matrix, and low-dimensional node embeddings. Let $G = (V, E, W)$ denote an undirected molecular network, where $V$ is the set of vertices (genes), $E$ is the set of edges (associations between genes), and $W$ is the set of edge weights (the strengths of the associations). $G$ can be represented as a weighted adjacency matrix $A_{i,j} = W_{i,j}$, where

$A \in R^{|V|\times|V|}$. $G$ can also be represented as an influence matrix, $F \in R^{|V|\times|V|}$, which can capture both local and global structure of the network. $F$ was obtained using a random walk with restart transformation kernel (8),

$$F = \alpha\left[I - (1 - \alpha)W_D\right]^{-1} \qquad \text{(eqn. 1)}$$

where, $\alpha$ is the restart parameter, $I$ is the identity matrix, and $W_D$ is the degree weighted adjacency matrix given by $W_D = AD^{-1}$, where $D \in R^{|V|\times|V|}$ is a diagonal matrix of node degrees. A restart parameter of 0.85 was used for every network.

$G$ can also be transformed into a low-dimensional representation through the process of node embedding. In this study we used the *node2vec* algorithm (9), which borrows ideas from the *word2vec* algorithm (10, 11) from natural language processing. The objective of *node2vec* is to find a low-dimensional representation of the adjacency matrix, $E \in R^{|V|\times d}$, where $d \ll V$. This is done by optimizing the following log-probability objective function:

$$E = \sum_{u\in V} log\left(Pr\left(N_s(u)|e(u)\right)\right) \qquad \text{(eqn. 2)}$$

where $N_s(u)$ is the network neighborhood of node $u$ generated through a sampling strategy $S$, and $e(u) \in R^d$ is the feature vector of node $u$. In *node2vec*, the sampling strategy is based on random walks that are controlled using two parameters *p* and *q*, in which a high value of *q* keeps the walk local (a breadth-first search), and a high value of *p* encourages outward exploration (a depth-first search). The values of *p* and *q* were both set to 0.1 for every network.

### Section 3: Processing gene set collections

The GenePlexus web-server uses two different gene set collections and the properties of these collections can be seen in Table S2. First, is a gene set collection that maps genes to various biological processes found in the Gene Ontology (12, 13). To build this gene set collection we retrieved gene to biological processes annotations from MyGene.info (6, 7) (downloaded on 2020-10-29) for any human gene that had an Entrez ID, where the annotations were subset to only include the following evidence codes; EXP, IDA, IPI, IMP, IGI, TAS, and IC. These annotations were propagated up the ontology, i.e. if a gene was annotated to a term, we then also annotated it to every ancestor term, where the ontology structure only included biological process terms. The other collection maps genes to various diseases. This mapping was downloaded directly from the DisGeNet database (14, 15) (downloaded on 2020-11-23), and we also propagated the gene-disease annotations to ancestor nodes using the Disease Ontology (16).

Each collection was also further processed separately for each network by first finding the intersection between the genes in a given network and the genes annotated to a term in the gene set collection. If the length of this intersection was between 10 and 200 the gene set was retained. After having gone through every term in the collection, we additionally keep track of all genes that are annotated to at least one term in this subset version of the gene set collection. This set of total genes is used when determining which genes to use as negative examples in the machine learning model.

**Table S2. Information on the gene set collections**. The last four columns reflect the fact each gene set collection is slightly different for every network and these values are presented as either a range, a median value, or number of genes in a union.

| gene set Collection | Network | Number of gene sets After Processing | gene set Size Range | Median gene set Size | Number of Unique Genes from Union of all gene sets |
|---|---|---|---|---|---|
| GO | BioGRID | 3692 | (10, 200) | 26 | 10,662 |
| | STRING | 3630 | (10, 200) | 26 | 10,643 |
| | STRING-EXP | 3622 | (10, 200) | 26 | 10,480 |
| | GIANT-TN | 3688 | (10, 200) | 26 | 10,897 |
| DisGeNet | BioGRID | 896 | (10, 198) | 26 | 6,126 |
| | STRING | 890 | (10, 199) | 26 | 6,055 |
| | STRING-EXP | 889 | (10, 199) | 26 | 5,977 |
| | GIANT-TN | 903 | (10, 196) | 26 | 6,231 |

## Section 4: Selecting positive and negative genes

The GenePlexus web-server uses a supervised machine learning model for predicting the association of all the genes in the network to the user supplied gene set. To build the classification boundary the model requires both positive and negative training examples. The positive set of genes is any gene from the user-supplied gene list that is able to be converted to an Entrez ID and found in the chosen network. The user can then choose if they want to define genes in the negative class based on one of two gene set collections, biological processes from the Gene Ontology (12, 13) or diseases from DisGeNet (14, 15), based on whether the input genes better represent a cellular process/pathway or a disease. GenePlexus then automatically selects the genes in the negative class by:

1. Consider the total pool of possible negative genes to be any gene that has an annotation to at least one of the terms in the selected gene set collection
2. Remove genes that are in the positive class.
3. For every term in a gene set collection, we perform a one-sided Fisher's exact test between the genes in the positive class and the genes annotated to the given term. If the p-value of the test is less than 0.05, all genes from the given term are also removed from the pool of possible negative genes.
4. The remaining genes in the pool of possible negative genes are used in the negative class. Note that most genes in the network are not contained in the positive class or negative class and are considered as part of the unlabeled class.

## Section 5: Supervised learning model

In GenePlexus, the supervised machine learning model uses the connections of a user chosen genome-scale molecular network as feature vectors. As described above, these feature vectors can be one of three representations; Adjacency, Influence and Embedding. The GenePlexus web-server uses logistic regression with l2-regularization as the supervised learning algorithm and is implemented using the python package *scikit-learn* (17). After training a model using the labeled genes, the trained model is used to classify all the genes in the chosen network, returning a prediction probability for these genes that is bounded between 0 and 1. The regularization parameter is set to 1.0 on the web-server.

## Section 6: Generating similarity scores

A unique feature of the GenePlexus web-server is proving some interpretation of the machine learning model trained on the user supplied gene set. This is done by comparing the weights of that trained model to the weights from thousands of other models pretrained on known gene sets of biological processes from the Gene Ontology and diseases from DisGeNet.

### *Section 6.1: Pre-training models*

The first step in this process is to train models for each known gene set. For each gene set in either the Gene Ontology or DisGenet collection a model is trained for every combination of network (BioGRID, STRING, STRING-EXP, GIANT-TN), feature type (Adjacency, Influence, Embedding) and way of selecting negatives (GO, DisGeNet) and the weights of these trained models are saved.

The next step is building up matrices that will be used for doing background correction of the final similarities presented on the web-server. To accomplish this, we generate a correction matrix, $C \in R^{|N| \times |T|}$, where $|N|$ is number of terms in the gene set collection that the user chose to build the negative class and $|T|$ is the number of terms in the gene set collection of the target table. For example, if the user chose to build the negative class based on biological processes in GO and the output result table on the web-server is displaying similarity of the user trained model to diseases in DisGeNet, then the rows of $C$ would correspond to biological process terms and the columns would correspond to disease terms. An element in the correction matrix is given by $C_{i,j} = S(w_{N_i}, w_{T_j})$, where $w$ is the vector of weights from a trained model and $S$ is a function that captures similarity between the two weight vectors. In this work, we use the cosine similarly as our similarity metric. A separate correction matrix is generated for all combinations of network, feature type, negative selection method and target table. We note that this requires training over >10,000 machine learning models where a model can have up to 25,689 weights and use thousands of training examples.

### *Section 6.2: Getting similarities to user trained model*

After the user submits a job, a custom machine learning model is trained. Once trained, the GenePlexus web-server computes the cosine similarity of the weights from the user model to the weights of each term in the target table, where $q \in R^{1 \times |T|}$ is given by $q_j = S(w_U, w_{T_j})$ and $w_U$ are the weights of the user model. This vector $q$ is then appended as the last row of the corresponding correction matrix, $C_{|N|+1,j} = q_j$. This is done separately for each target table.

### *Section 6.3: Background correction*

The background correction is done in two parts. First, a z-score is calculated across all scores for the user genes, which is given by,

$$z_{q_j} = max\left(0, \frac{C_{|N|+1,j} - \mu(|N|+1)}{\sigma(|N|+1)}\right),$$

Where $\mu(|N| + 1)$ and $\sigma(|N| + 1)$ are the mean and standard deviation calculated across the $|N| + 1$ row of $C$, respectively. Additionally, a z-score is calculated to correct for any bias in the negative gene selection, which is given by,

$$z_{T_j} = max\left(0, \frac{C_{|N|+1,j} - \mu(j)}{\sigma(j)}\right),$$

Where $\mu(j)$ and $\sigma(j)$ are the mean and standard deviation calculated across the $j^{th}$ column of $C$, respectively. The final scores presented on the GenePlexus web-server are the l2-norm of the above z-scores given by,

$$z_j = \sqrt{z_{q_j}^2 + z_{T_j}^2}.$$

## References


1. Stark,C., Breitkreutz,B.-J., Reguly,T., Boucher,L., Breitkreutz,A. and Tyers,M. (2006) BioGRID: a general repository for interaction datasets. *Nucleic Acids Res.*, **34**, D535–D539.
2. Oughtred,R., Stark,C., Breitkreutz,B.-J., Rust,J., Boucher,L., Chang,C., Kolas,N., O'Donnell,L., Leung,G., McAdam,R., *et al.* (2019) The BioGRID interaction database: 2019 update. *Nucleic Acids Res.*, **47**, D529–D541.
3. Szklarczyk,D., Gable,A.L., Lyon,D., Junge,A., Wyder,S., Huerta-Cepas,J., Simonovic,M., Doncheva,N.T., Morris,J.H., Bork,P., *et al.* (2019) STRING v11: protein–protein association networks with increased coverage, supporting functional discovery in genome-wide experimental datasets. *Nucleic Acids Res.*, **47**, D607–D613.
4. Greene,C.S., Krishnan,A., Wong,A.K., Ricciotti,E., Zelaya,R.A., Himmelstein,D.S., Zhang,R., Hartmann,B.M., Zaslavsky,E., Sealfon,S.C., *et al.* (2015) Understanding multicellular function and disease with human tissue-specific networks. *Nat. Genet.*, **47**, 569–576.
5. Huang,J.K., Carlin,D.E., Yu,M.K., Zhang,W., Kreisberg,J.F., Tamayo,P. and Ideker,T. (2018) Systematic Evaluation of Molecular Networks for Discovery of Disease Genes. *Cell Syst.*, **6**, 484-495.e5.
6. Xin,J., Mark,A., Afrasiabi,C., Tsueng,G., Juchler,M., Gopal,N., Stupp,G.S., Putman,T.E., Ainscough,B.J., Griffith,O.L., *et al.* (2016) High-performance web services for querying gene and variant annotation. *Genome Biol.*, **17**, 91.
7. Wu,C., MacLeod,I. and Su,A.I. (2013) BioGPS and MyGene.info: organizing online, gene-centric information. *Nucleic Acids Res.*, **41**, D561–D565.
8. Leiserson,M., Vandin,F., Wu,H.-T., Dobson,J., Eldridge,J., Thomas,J., Papoutsaki,A., Kim,Y., Niu,B., McLellan,M., *et al.* (2015) Pan-Cancer Network Analysis Identifies Combinations of Rare Somatic Mutations across Pathways and Protein Complexes. *Nat. Genet.*, **47**, 106–114.
9. Grover,A. and Leskovec,J. (2016) node2vec: Scalable Feature Learning for Networks. In *Proceedings of the 22nd ACM SIGKDD International Conference on Knowledge Discovery and Data Mining - KDD '16*. ACM Press, San Francisco, California, USA, pp. 855–864.
10. Mikolov,T., Sutskever,I., Chen,K., Corrado,G. and Dean,J. (2013) Distributed Representations of Words and Phrases and their Compositionality. *ArXiv13104546 Cs Stat.*
11. Mikolov,T., Chen,K., Corrado,G. and Dean,J. (2013) Efficient Estimation of Word Representations in Vector Space. *ArXiv13013781 Cs.*
12. The Gene Ontology Consortium (2019) The Gene Ontology Resource: 20 years and still GOing strong. *Nucleic Acids Res.*, **47**, D330–D338.
13. Ashburner,M., Ball,C.A., Blake,J.A., Botstein,D., Butler,H., Cherry,J.M., Davis,A.P., Dolinski,K., Dwight,S.S., Eppig,J.T., *et al.* (2000) Gene ontology: tool for the unification of biology. The Gene Ontology Consortium. *Nat. Genet.*, **25**, 25–29.
14. Piñero,J., Bravo,À., Queralt-Rosinach,N., Gutiérrez-Sacristán,A., Deu-Pons,J., Centeno,E., García-García,J., Sanz,F. and Furlong,L.I. (2017) DisGeNET: a comprehensive platform integrating information on human disease-associated genes and variants. *Nucleic Acids Res.*, **45**, D833–D839.
15. Piñero,J., Queralt-Rosinach,N., Bravo,À., Deu-Pons,J., Bauer-Mehren,A., Baron,M., Sanz,F. and Furlong,L.I. (2015) DisGeNET: a discovery platform for the dynamical exploration of human diseases and their genes. *Database*, **2015**.
16. Schriml,L.M., Mitraka,E., Munro,J., Tauber,B., Schor,M., Nickle,L., Felix,V., Jeng,L., Bearer,C., Lichenstein,R., *et al.* (2019) Human Disease Ontology 2018 update: classification, content and workflow expansion. *Nucleic Acids Res.*, **47**, D955–D962.
17. Pedregosa,F., Varoquaux,G., Gramfort,A., Michel,V., Thirion,B., Grisel,O., Blondel,M., Prettenhofer,P., Weiss,R., Dubourg,V., *et al.* (2011) Scikit-learn: Machine Learning in Python. *J. Mach. Learn. Res.*, **12**, 2825−2830.
